# Transcriptomic heterogeneity of cultured ADSCs corresponds to embolic risk in the host

**DOI:** 10.1016/j.isci.2022.104822

**Published:** 2022-08-04

**Authors:** Kaijing Yan, Jinlai Zhang, Wen Yin, Jeffrey N. Harding, Fei Ma, Di Wu, Haibo Deng, Pengfei Han, Rui Li, Hongxu Peng, Xin Song, Y. James Kang

**Affiliations:** 1 Regenerative Medicine Research Center, Sichuan University West China Hospital, Chengdu, Sichuan 610044, China; 2 Stem Cell Biology Laboratory, Tasly Pharmaceutical Co. Ltd, Tianjin 300410, China

**Keywords:** Stem cells research, computational bioinformatics, transcriptomics

## Abstract

Stem cell therapy emerges as an effective approach for treating various currently untreatable diseases. However, fatal and unknown risks caused by their systemic use remain to be a major obstacle to clinical application. We developed a functional single-cell RNA sequencing (scRNA-seq) procedure and identified that transcriptomic heterogeneity of adipose-derived stromal cells (ADSCs) in cultures is responsible for a fatal embolic risk of these cells in the host. The pro-embolic subpopulation of ADSCs in cultures was sorted by gene set enrichment analysis (GSEA) and verified by a supervised machine learning analysis. A mathematical model was developed and validated for the prediction of embolic risk of cultured ADSCs in animal models and further confirmed by its application to public data. Importantly, modification of culture conditions prevented the embolic risk. This novel procedure can be applied to other aspects of risk assessment and would help further the development of stem cell clinical applications.

## Highlights

• Transcriptomic heterogeneity of ADSCs can be detected by scRNA-seq
• A functional scRNA-seq method was developed to identify pro-embolic ADSC subpopulations
• A mathematical model was built and validated for the prediction of embolic risk of ADSCs
• Embolic risk of ADSCs can be prevented by the manipulation of cell culture conditions

## Graphical abstract

**Figure:**
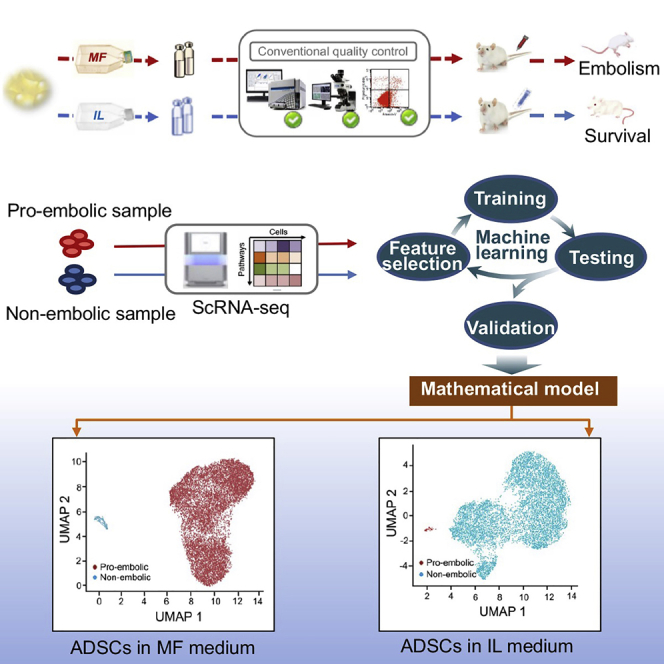

## Introduction

Stem cell therapies have demonstrated an unparalleled potential in restoring tissue function and treating the root cause of degenerative diseases (Trounson and McDonald, 2015), but their clinical applications face unprecedented challenges of safety issues such as embolism, tumorigenicity, and immunogenicity (Amariglio et al, 2009.; Mathe et al., 1967; Woodard et al., 2000). Systemic infusion of stem cells is critical to achieve the efficacy of stem cell therapy (Kallmeyer et al., 2020; Yan et al., 2021); however, there is increasing evidence that demonstrates the fatal risk resulting from embolism caused by stem cell infusion in both animal and human studies (Cyranoski, 2010; Tatsumi et al., 2013).

Stem cells isolated from their naturally occurring niches undergo stress responses to the new environment during the process of propagation (Discher et al., 2009). Embolism-related genes are differentially expressed among individual stem cells from the same origin under culture conditions (George et al., 2018; Oeller et al., 2018). It is reasonable to assume that in the whole population, the percentage of stem cells expressing pro-embolic factors correlates with their pro-embolic activity (Christy et al., 2017; George et al., 2018; Oeller et al., 2018). It was reported that cell culture protocols profoundly change the gene expression profiles among stem cells and thereby affect their heterogeneity (Sotiropoulou et al., 2006; Winkel et al., 2020).

Several guidelines and recommendations have been proposed to define stem cell populations, aiming at ensuring the reproducibility of stem cell preparations (Dominici et al., 2006; Galipeau et al., 2016; FDA, 2011; ISSCR, 2021). However, the safety issue of stem cell therapy remains unaddressed. The US-FDA Cellular and Tissue Therapies Branch (CTTB) states that the major challenges of the safety issue lie in the inadequate markers predictive of cell state/fate and poor understanding of how cells interact with their microenvironment (https://www.fda.gov/media/140359/download).

Therefore, we took the present study to specifically address (1) how to detect heterogeneity of tissue-derived multipotent stromal cells (MPSCs) in cultures for quality control analysis of their propagation; (2) developing an analytical procedure to identify subpopulations of risk possessing MPSCs, such as embolic risk in the host; and (3) establishing a prediction model for the probability of deleterious results caused by subpopulations of risk-possessing MPSCs in the host (animal models).

## Results

### Embolism is currently an unpredictable fatal risk

Human adipose-derived stromal cells (hADSCs) have been shown to have a high efficacy in recovering acute brain damage from ischemic insult (Gutiérrez-Fernández et al., 2015). We cultured these cells in commercially available media, supplemented with fetal bovine serum (FBS). These cells often caused embolism after their infusion, as reported by several laboratories (Furlani et al., 2009; Sugiyama et al., 2018; Tatsumi et al*.*, 2013). Several studies have shown that lowering the concentration of FBS in cultures may reduce the embolic risk of stem cells (Dreher et al., 2013; Gutiérrez-Fernández et al., 2015). Thus, we acquired a chemically defined medium (IL-medium, Innolife Co) and found that the same hADSCs cultured in the IL-media without FBS supplementation were embolic risk-free in mouse models.

**Figure 1:**
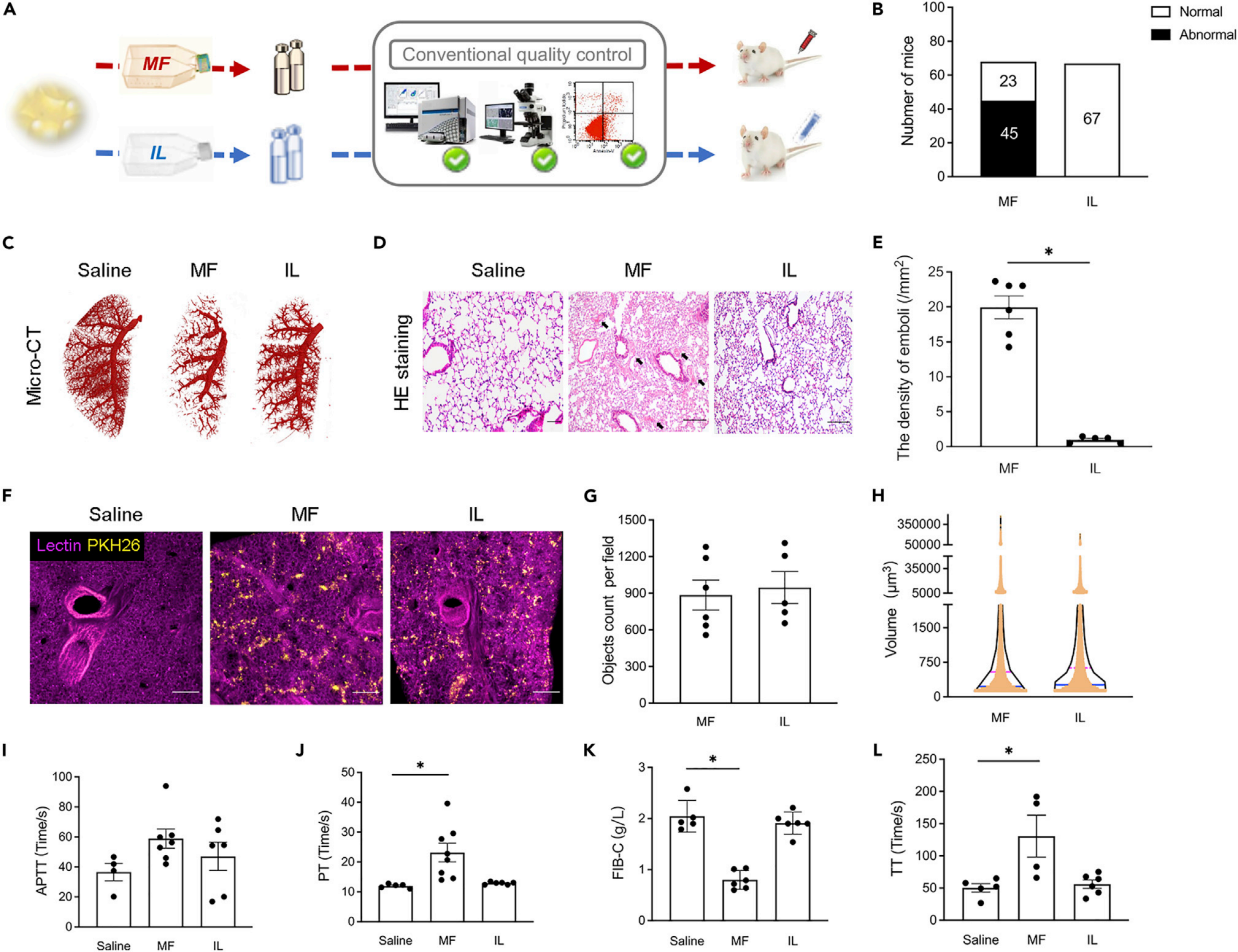
Analysis of embolism after infusion of stem cells cultured in MF-media or in IL-media (A) Human ADSCs obtained from the same donor by the same procedure, but cultured in MF-media or IL-media before infusion into mice. (B) Numerical difference in mice showing typical symptoms of pulmonary embolism induced by hADSCs obtained from MF-media or IL-media. (C) Computed tomography of mouse lungs infused with hADSCs in comparison to saline control. (D) Histopathological examination of embolism formation in the lungs postinfusion of hADSCs cultured in either MF or IL medium. Black arrows indicate phlebothrombosis; Bar = 200 μm. (E) The density of emboli found in each 10× visual field (1 × 10^6^cells per group); ∗p < 0.05. (F) PKH26 + hADSCs and lectin + vessels in the lungs of mice infused with saline or hADSCs cultured in MF-media or in IL-media. (G) Number of PKH26 + hADSCs found in each 10× visual field (1×10^6^ cells per group). (H) Detected volume of PKH26 + hADSCs in each 1.0 mm of lung tissue obtained from mice after infusion of hADSCs cultured in MF-media or in IL-media. (I–L) Examination of four blood coagulation indexes (I) APTT, Activated partial thromboplastin time, (J) PT, Prothrombin time, (K) FIB-C, Fibrinogen-C, (L) TT, Thrombin time; ∗p < 0.05. Each data point represents a biological replicate; data are mean ± s.e.m. Images are representative of six biological replicates. Data were analyzed using Student’s unpaired two-tailed t-test when comparing two conditions and ANOVA when comparing multiple conditions.

To elucidate the development of heterogeneity of hADSCs during their propagation under different cultural conditions, we compared the differences between the cells prepared in IL-media and those in FBS-containing media. As shown in Figure 1A, we cultured hADSCs from the same donor in MF (αMEM + FBS) media or in IL-media and infused these cells into mice. We found that among 68 mice infused with hADSCs obtained from the MF-media, 45 mice developed typical pulmonary embolism symptoms within 1 min after the infusion, as manifested by breathlessness and convulsion, while there were no abnormalities in any of the 67 mice infused with hADSCs obtained from the IL-media (Figure 1B). Pulmonary angiography revealed a large number of blockages in the pulmonary vasculatures (Figure 1C), and histological staining of the lung showed a significant number of venous clots in the mice infused with hADSCs from the MF-media (Figures 1D and 1E). In contrast, the hADSCs obtained from the IL-media did not cause any of the above-mentioned adverse effects (Figures 1B–1E).

In order to clarify whether the formation of the thrombus is caused by hADSCs aggregates, we labeled hADSCs with fluorescent PKH26, tracing these cells after their infusion. By counting the number of PKH26-positive cells in the lung, we found that there was no significant difference between the mice infused with hADSCs obtained from the MF-media versus from the IL-media (Figures 1F–1H), indicating that hADSCs retention in the lung is not the major reason of pulmonary embolism.

We next examined if the activation of coagulation is responsible for the pulmonary embolism induced by hADSCs obtained from the MF-media. We found that there was no significant change in the activated partial thromboplastin time (APTT), but an increase in the prothrombin time (PT) and the thrombin time (TT), and a decrease in the fibrinogen-C (FIB-C) concentration (Figures 1I–1L), in the mice receiving hADSCs obtained from the MF-media, indicating the activation of the coagulation reaction. In contrast, we did not observe any of these changes in the mice infused with hADSCs obtained from the IL-media (Figures 1I –1L). These results demonstrate that the hADSCs propagated under different culture conditions undergo different biological processes, or develop different lineages. In particular, MF-media enabled a pro-coagulating reaction in the hADSCs. What are the differences between the ADSCs prepared under different culture conditions?

We followed currently consented quality control criteria of the International Society for Cellular Therapy (ISCT) for intravenous infusion to identify the difference between the two batches of hADSCs. To our surprise, there were no distinct differences between the two batches of cells (Figures S1 and S2). Both of these cells met the consented quality control criteria for intravenous infusion. Therefore, the currently consented criteria for quality control of stem cell preparations do not reveal the lineage difference that actually exists in these propagated hADSCs under different culture conditions. This limitation of current quality control criteria was also identified in other studies (Robey, 2017).

### Stem cell heterogeneity revealed by scRNA-seq

The unique scRNA-seq technique for the transcriptomic profiling of tens of thousands of cells at a single-cell resolution enables us to uncover cellular heterogeneity and predict cell fate (Tang et al., 2009; Weinreb et al., 2020). We used this technique to define the differences between the hADSCs prepared in different cultural media. We used a BD Rhapsody-based protocol through barcoding with unique molecular identifiers to start the initial screening process. We sequenced 15,779 and 9,141 cells obtained from the MF-media and the IL-media, respectively, and achieved, on average, 54.70 K and 51.14 K reads per cell, respectively. The sequencing saturation rate was 78.35 and 81.37%, resulting in 4858 and 4815 genes per cell, respectively. After quality filtering for the number of detected genes and mitochondrial read counts, duplicates were assessed, and unique transcripts were normalized for total read depth. We analyzed the heterogeneity of cells from different samples, phases, and donors, and integrated samples using a uniform manifold approximation and projection (UMAP) (Figures S3A–S3E). There were seven distinct clusters that were identified in both cell populations (Figure S3F). The proportions of the cells distributed in these clusters were different: hADSCs obtained from the MF-media were predominantly sorted in clusters 0, 1, and 2, while hADSCs from the IL-media were in clusters 1, 2, and 3 (Figures S3G–S3I). Therefore, the scRNA-seq analysis, indeed, identified the heterogeneity of stem cells prorogated under different culture conditions. However, this analysis did not tell us the specific distinction in embolic risk between the two batches of cells or justify the reason why hADSCs obtained from the IL-media did not cause embolism.

### Functional scRNA-seq assessment for embolic risk

#### Functional clustering based on canonical pathways

**Figure 2:**
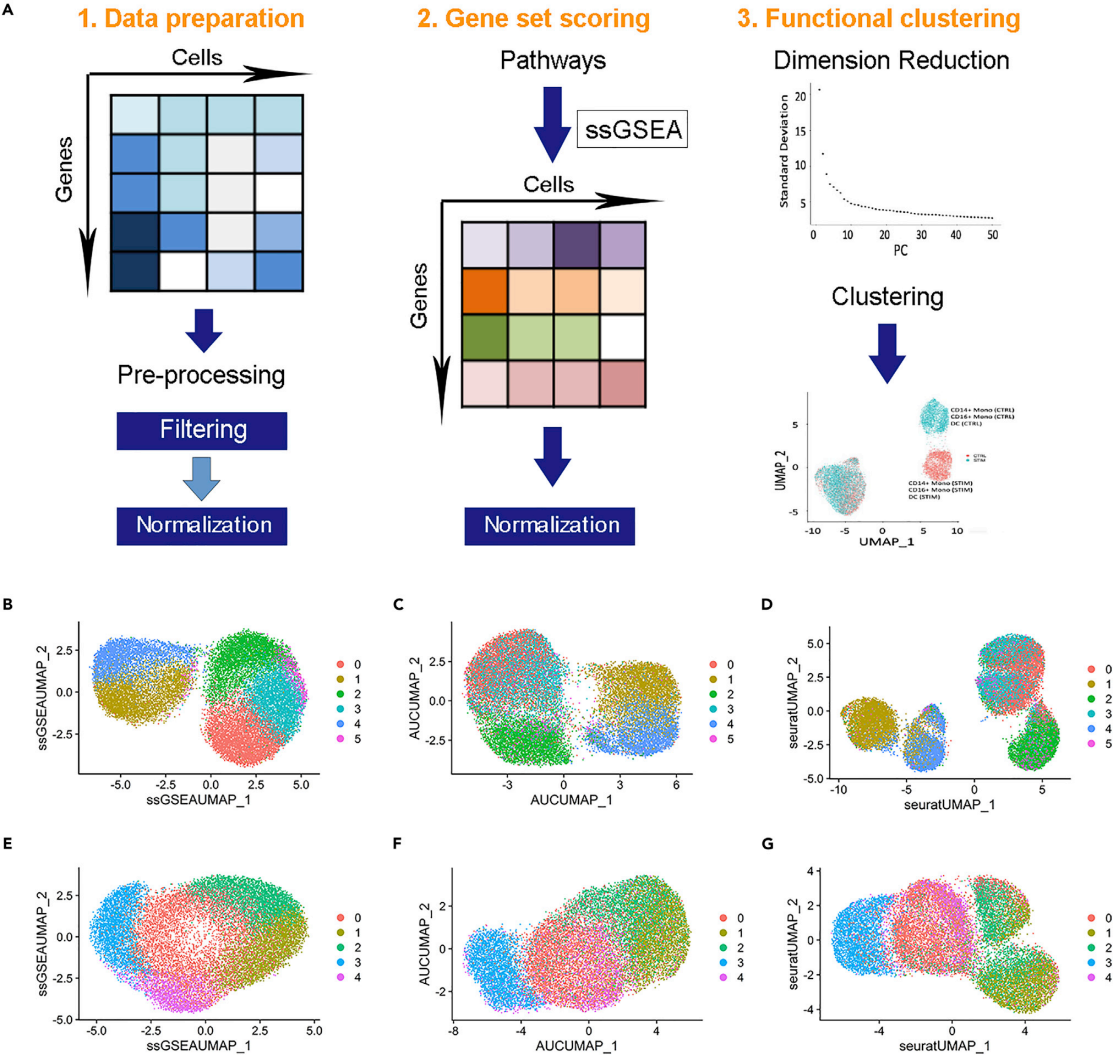
Detection of heterogeneity of hADSCs by a new functional clustering procedure (A) Schematic diagram of the development of the functional clustering procedure. (B and E) Functional clustering using the gene set enrichment analysis algorithm from ssGSEA of hADSCs obtained from MF-media (B) shows 6 clusters, and from IL-media (E) showing 5 clusters. (C and F) Functional clustering using the area under curve algorithm from AUCell of the hADSCs above shows 6 clusters for the cells from the MF-media (C) and 5 clusters for the cells from the IL-media (F). (D and G) Functional clustering using the Seurat module score algorithm (Seurat) of the hADSCs above showing 6 clusters for the cells from the MF-media (D) and 5 clusters for the cells from the IL-media (G). There were 28,732 hADSCs analyzed in total.

The evolution of heterogeneity of hADSCs during their propagation would be the result of differential responses of individual cells to the microenvironment of culture conditions. In scRNAseq analysis, gene expression of single cells can be transformed into pathway activity to uncover potential mechanisms of cells and predict cell potential phenotypic fate (Ding et al., 2019; Ramirez et al., 2020; Wang et al., 2020). The pro-embolic cell subpopulation would be composed of the cells in which the pro-embolic pathways were activated in response to the triggering ligands present in the media. Therefore, the analysis of pro-embolic pathways could help identify pro-embolic cells. To identify this subpopulation, we first establish a functional clustering procedure through gene set enrichment analysis (GSEA) for canonical pathways (Figure 2A). The rationale is that if any pathway is activated during the propagation, the expression of the genes involved in that pathway would be up-regulated and the cell will be clustered in the same group of cells with the same set of gene expressions.

We found there were some distinct clusters, each involving cells that would be activated in some particular pathways, as shown in Figures 2B–2E. There were 6 relatively distinct clusters among the cells obtained from MF-media, but 5 clusters among the cells from the IL-media. To verify this result, we used two other testing procedures, Seurat and AUCell, to analyze the same dataset and to reduce possible analytical bias from a single procedure. We found the same functional clusters, i.e., 6 clusters among the cells from the MF-media (Figures 2C and 2D) and 5 from the IL-media (Figures 2F and 2G), revealed by the latter two analytical procedures. This result indicates that once MPSC is triggered by a ligand in the microenvironment, its lineage would be evolved and progressed, and the cell would not respond to other ligands. If this is true, the cells that are triggered to become a pro-embolic subpopulation would be distinguishably separated from other cells, being sorted into a distinct cluster.

#### Identification of pro-embolic human adipose-derived stromal cell cluster

**Figure 3:**
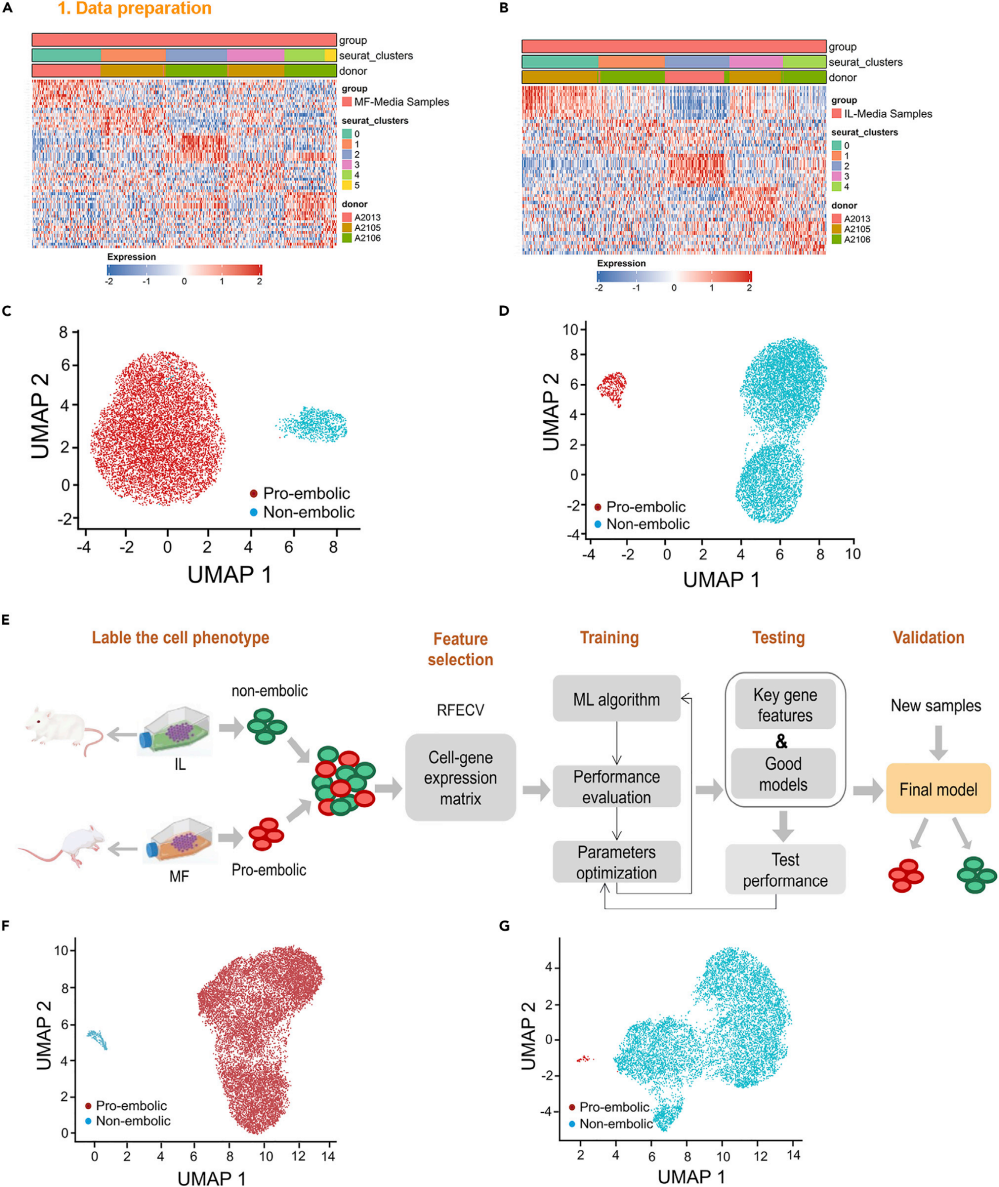
Detection of pro-embolic hADSC subpopulation by a new functional clustering procedure and a machine learning algorithm (A) Heatmap of top differentially expressed genes in functional clusters of hADSCs cultured in the MF-media. (B) Heatmap of top differentially expressed genes in functional clusters of hADSCs cultured in the IL-media. (C) Supervised clustering of a subset of pro-embolic hADSCs cultured in the MF-media based on known genes involved in the embolism-related pathways. (D) Supervised clustering of a subset of pro-embolic hADSCs cultured in the IL-media based on known genes involved in the embolism-related pathways. (E) Schematic diagram of the machine learning algorithm training and testing to predict potential pro-embolic cells. (F) Supervised clustering of hADSCs cultured in the MF-media based on 13 key genes identified by the machine learning algorithm. (G) Supervised clustering of hADSCs cultured in the IL-media based on 13 key genes identified by the machine learning algorithm.

We next performed the heatmap analysis of genes differentially expressed in different clusters and found that the genes involved in pro-embolic pathways, including complement and coagulation cascades, intrinsic pathway of fibrin clot formation, extrinsic pathway of fibrin clot formation, and common pathway of fibrin clot formation were up-regulated in clusters 0 and 3 in cells from either MF-media or IL-media (Figures 3A and 3B, S4, and Table S4). We then pooled the cells in clusters 0 and 3 and re-analyzed these cells by the GSEA procedure based on the genes defined from the heatmap analysis. We found that the majority of cells obtained from the MF-media along with an up-regulation of pro-embolic genes including *SERPINE1*, *F3, and CD55,* were sorted into a pro-embolic cluster. There was a very small proportion of the cells along with an up-regulation of anti-embolic genes such as *C1R*, *CFH*, *F2R,* and others, were in the non-embolic cluster (Figure 3C and Table S4). In contrast, the cells from the IL-media were inversely distributed (Figure 3D). These pro- and anti-embolic genes are intimately involved in the coagulation-related pathways, especially in the complement and coagulation cascades and common pathways of fibrin clot formation (Figures S5A and S5B).

We next used a supervised machine learning method to verify the GSEA result. We first assigned the cells into two categories (pro-embolism or non-embolism) based on animal experiment outcomes, i.e., cells from MF-media were categorized as pro-embolic cells and cells from IL-media were categorized as non-embolic cells.

We used the eigenvectors from the gene expression values of cells to develop predictive models for embolism via a training set and a series of test sets. We first identified 13 key genes which could improve the accuracy of 10-fold cross-validation to 1 using recursive feature elimination (RFE) (Figures S6A and S6B). Then, we trained candidate models with different complexity parameter values (C value) to guarantee that the final model would have a high training accuracy and good performance in the test sets using the support vector machine (SVM) algorithm (Figures 3 E, S6A and S6B). Finally, we determined the candidate model with C = 0.2 as the final model which performed best in all test sets with prediction accuracy reaching 1, 1, 0.97, and 0.95, respectively (Table S1). The 13 key genes in the final model were *IL6*, *TAGLN*, *LRRC17*, *CRIM1*, *SERPINE2*, *FOS*, *ARL4C*, *LUM*, *THBS2*, *EFEMP1*, *BCYRN1,* and *FBLN5*. As shown in Figures 3F and 3G, the machine learning analysis revealed two distinct clusters, almost an identical result to that of the GSEA analysis.

#### Mathematical model to predict embolic risk

In order to quantitatively define the embolic risk of hADSCs, we developed a mathematical model to calculate the embolic risk score (RS) of each cell based on the expression of the identified 13 key genes and the weighing coefficient vector (**w**) from the final predictive model. Briefly, we input a training set T = {(x_1_, y_1_), (x_2_, y_2_), …, (x_n_, y_n_), where x_i_ is the eigenvector representing cell i, y_i_ ∈ {+1, −1) representing embolic and non-embolic label, i = 1, 2, … n, then we calculated **w** which is the normal vector of the optimal separating hyperplane for classification using Equation (1):

(Equation 1) $$w=\sum_{\text{i}=1}^{\text{N}}{\text{α}}_{\text{i}}^{\text{∗}}{\text{y}}_{\text{i}}\left({\text{x}}_{\text{i}}\cdot {\text{x}}_{\text{j}}\right)$$

Where ${\text{α}}_{\text{i}}^{\text{∗}}$ is a component of $\alpha^{*}$ which is the Lagrange multiplier calculated using the sequential minimal optimization algorithm. We can distinguish the pro-embolic and non-embolic cells by decision function (Equation 2):

(Equation 2) $$\text{f}\left(\text{x}\right)=\text{sign}\left({\text{w}}^{\text{T}}\;\text{x}+\text{b}\right)$$

where **x** is the eigenvector representing a cell, b is the distance of hyperplane from the initial point of zero, the sign function is a hard limiting function that does not infer any source information (i.e., donor, passage number, and expansion protocol) of the sample, which can largely influence differentiation potential of hADSCs (Fan et al., 2020). We finally use the monotonically increasing continuous S-type function f(x) = 1+e^-x^ to calculate RS of pro-embolic cells as function (Equation 3):

(Equation 3) $$\text{RS }=1+e^{-\sum_{i=1}^{n}W_{i}\;*\;G_{i}}$$

Where *Wi* is the weighted coefficient of the *i*th gene in weighing coefficient vector **w** determined by the prediction model function, as shown above, *Gi* is the expression of the *i*th gene in this cell, and n is the number of key genes. The value of RS ranges from 0 to ∞ with a small risk score indicating a non-embolic cell and a larger risk score indicating a potential embolic cell. After obtaining the RS of each cell, we use a ROC curve analysis to determine the RS threshold of pro-embolic and non-embolic cells in test samples. From the ROC curve, the thresholds of the four test sets were defined to be 2.131, 2.131, 2.048, and 3.368, respectively. The specificity and sensitivity of using the thresholds to distinguish cell embolism are more than 0.96 in each test dataset (Figures 3E and S6C–S6G).

#### Validation of the embolism risk predictive model

We performed a series of experiments infusing hADSCs prepared from different cultures containing varying concentrations of FBS. These cells were analyzed and the proportion of pro-embolic cells in the entire population was predetermined by the predictive model and verified by GSEA. We found that with an increase in FBS concentrations in cultures, the proportion of pro-embolic cells likewise increased. The predictive model indicates that when the subpopulation of the pro-embolic stem cells reached 13.16%, an infusion of 1 million cells per mouse will inevitably cause pulmonary embolism, as verified by animal studies (Figure S6H).

To determine the applicability of the mathematic model in other scenarios, we analyzed the single-cell sequencing data of a public hADSCs sample. The cells were cultured in low-glucose Dulbecco’s Modified Eagle’s Medium (DMEM) supplemented with 15% FBS (Liu et al., 2019). We found that 97.3% of the cells are pro-embolic (Figure S6I), which is proportional to that (87.8%) of the cells prepared from the MF-media (10% FBS) in the present study.

## Discussion

Here, we developed a functional scRNA-seq procedure and a predictive model for the assessment of embolic risk of hADSCs. Embolism caused by ADSC infusion is a major obstacle to clinical applications of cell therapy. Cell propagation is the prerequisite for the clinical application of ADSCs and other MPSCs or mesenchymal stem cells (MSCs) in treating multiple currently untreated diseases. In this process, cells are exposed to an altered microenvironment under different culture conditions after they are isolated from their naturally occurring niches. Individual cells would differentially respond to the microenvironment, thus evolving different lineages in the same population from the same origin, constituting the heterogeneity of the propagated cells.

How to detect the heterogeneity of MPSCs or MSCs is a major challenge in the safety assessment for cellular therapy in clinical applications. A classic unsupervised scRNA-seq analysis, indeed, identified the heterogeneity of ADSCs in cultures. It appears that once an MPSC or MSC cell is triggered by a particular ligand in the cultures, it would commit to a specific lineage through the activation of a particular pathway. This lineage evulsion of a single cell is the result of its transcriptomic shift in response to a stimulus in culture and thus makes it heterogenic to the entire population. Therefore, the use of the scRNA-seq analysis in this study clearly identified the cell subpopulations with a transcriptomic shift, effectively detecting the heterogeneity of ADSCs in cultures.

The understanding of how MPSCs or MSCs interact with their microenvironment is of significant clinical relevance. In the present study, we observed that the activation of the gene sets of ADSCs in cultures only involves a small number of particular canonical pathways, indicating that the progression of a single cell lineage in cultures would not be disturbed once the lineage is evolved, although there are thousands of canonical pathways in a single cell. In this context, the functional clustering procedure developed here should provide a valuable tool to identify specific risk subpopulations based on their transcriptomic profile, as approved by the detection of embolic risk of ADSCs in the present study. Therefore, as long as the gene set of a particular pathway is known, a particular cell subpopulation with the evolved lineage would be identified following the same principle of functional clustering. That said, other aspects of safety issues, such as tumorigenicity and immunogenicity, of MPSCs or MSCs would be resolved in the future.

A critical issue in the assessment of embolic risk is how many pro-embolic cells in a whole population are of high risk? The mathematical model developed here specifically addressed this question. We verified this predictive model through experimental animal models and defined the quantity threshold for the likelihood of embolism caused by the propagated ADSCs. This model is also applicable to analyzing public data, as demonstrated in the present study. Importantly, the predictive model indicates that by the modification of culture conditions, the pro-embolic risk is preventable.

In our preliminary studies, we have used bone marrow mesenchymal stem cells (BMSCs) for the screening test and found that the phenomenon characterized by the ADSCs here holds true for BMSCs. Therefore, we speculate that the functional scRNAseq procedure and mathematical model developed in the present study are applicable to other MPSCs and MSCs. In the clinical setting, it is important to define the consistency of efficacy, as well as the safety assurance, of MPSCs and MSCs prepared under different culture conditions. The model presented in the present study would help to address these issues. Meanwhile, we would recommend MPSCs and MSCs used for clinical use should be propagated in well-characterized, serum-free, chemically defined media. The principle of the functional scRNAseq procedure would be applicable to the characterization of clinically safe culture conditions.

### Limitations of the study

This work focuses on developing a novel functional scRNA-seq procedure to assess risks derived from the heterogeneity of ADSCs after their propagation. The framework was established but data enrichment is needed for further development of the predictive model for more general application to risk the assessment of MPSCs and MSCs in clinical application. In addition, mechanisms by which the heterogeneity evolves during the propagation of ADSCs in cultures need to be deeply probed in order to develop intervention procedures to avoid the risks.

## STAR★Methods

### Key resources table

**Table:** 

REAGENT or RESOURCE	SOURCE	IDENTIFIER
**Antibodies**		

Lectin from *Triticum vulgaris* (wheat)	Sigma	Cat#L4895; Lot#049M4006V
PKH26 Red Fluorescent Cell Linker Mini Kit	Sigma	Cat#MINI26-1KT; Lot#MKCM8734
FITC Mouse IgG1, k, Iso-type Ctrl (FC)	BioLegend	Cat#400110; Lot#B283622; RRID: AB_2861401
FITC anti-human CD34	BioLegend	Cat#343504; Lot#B252495; RRID: AB_1731852
FITC anti-human CD45	BioLegend	Cat#304006; Lot#B293670; RRID: AB_314394
FITC anti-human CD11b	BioLegend	Cat#301330; Lot#B272326; RRID: AB_2561703
FITC anti-human HLA-DR	BioLegend	Cat#307604; Lot#B275368; RRID: AB_314682
FITC anti-human CD73	BioLegend	Cat#344016; Lot#B288647; RRID: AB_2561809
FITC anti-human CD90	BioLegend	Cat#328108; Lot#B291322; RRID: AB_893429
APC Mouse IgG1, k, Iso-type Ctrl	BioLegend	Cat#400120; Lot#B299375; RRID: AB_2888687
APC anti-human CD19	BioLegend	Cat#363006; Lot#B299554; RRID: AB_2564128
PE Mouse IgG1, k, Iso-type Ctrl (FC)	BioLegend	Cat#400114; Lot#B278607; RRID: AB_326435
PE anti-CD105 (Endoglin)	BioLegend	Cat#800504; Lot#B289657; RRID: AB_2629655

**Chemicals, peptides, and recombinant proteins**		

0.9% Saline	Shijiazhuang SiYao	Cat#H13023200
Collagenase, Type I	Gibco	Cat#17100-017
αMEM	Gibco	Cat#41061
Fetal Bovine Serum	NCT	Cat#NCT-HK023
IL media	Innolife	Cat#09-RM-HH01A
Phosphate-Buffered Saline(1X)	Cytiva	Cat#SH30256.01
Dulbecco’s Phosphate-Buffered Saline	Gibco	Cat#14190144
AO/PI Viability Assay	DeNovix	Cat#CD-AO-PI-1.5
1×Tryple Express	Gibco	Cat#12604-021
CryoPur-D	Origen	Cat#CD-50
Microfil	Flow Tech Inc	Cat#MV-120
Hematoxylin-eosin (H&E) dye	Biosharp	Cat#BL700B
Ethanol absolute	Tianjin Fengchuan	Cat#XK 13-011-10003
APC Beads	BD Biosciences	Cat#340487
DMEM/F12 without phenol	Gibco	Cat#11039-021
4% Paraformaldehyde solution	Beyotime	Cat#P0099-500 mL
10% formalin Neutral Fixative	YuLu Experiment Equipment	Lot#210105
Dipping Paraffin and Dewaxing Solution (eco-friendly)	YuLu Experiment Equipment	Lot#210105
Permount TM Mounting Medium	Tianjin Zhiyuan Reagent co., Ltd	N/A
Tryptic Soy Broth(TSB)	Beijing Sanyao Science&Technology Development Co.	Cat#16621; Lot#20201210
Fluid Thioglycollate Medium	Beijing Sanyao Science&Technology Development Co.	Cat#16612; Lot#20210111
Steritailin	TAILIN	Cat#PY330; Lot#2021032801

**Critical commercial assays**		

Cell cycle and apoptosis detection kit (containing RNase A)	4A Biotech	Cat#FXP0211
Annexin V-FITC Apop kit	Invitrogen	Cat#BMS500FI-300
3-color kit	BD Biosciences	Cat#340486
Cell Counting Kit-8	MCE	Cat#HY-K0301
Martius Scarlet Blue (MSB) stain for fibrin	GENMED	Cat#GMS80125.1
Osteogenic Differentiation Medium for Human Adipose-derived Mesenchymal Stem Cells	Cyagen	Cat#HUXMD-90021
Adipogenic Differentiation Medium for Human Adipose-derived Mesenchymal Stem Cells	Cyagen	Cat#HUXMD-90031
Chondrogenic Differentiation Medium for Human Bone Marrow Mesenchymal Stem Cells	Cyagen	Cat#HUXMD-90041
Cartridge Reagent Kit	BD Biosciences	Cat#633731
Cartridge Kit	BD Biosciences	Cat#633733
cDNA Kit	BD Biosciences	Cat#633773
Whole Transcriptome Analysis (WTA) Amplification Kit	BD Biosciences	Cat#633801
Agilent DNA 7500 Kit	Agilent Technologies	Cat#5067-1506

**Deposited data**		

Human adult adipose tissue	Tianjin first central hospital	http://www.tj-fch.com

**Experimental models: Organisms/strains**		

Mouse: NCG	GemPharmatech	N/A

**Software and algorithms**		

ImageJ	National Institutes of Health, USA	https://imagej.nih.gov/ij
Prism	GraphPad	https://www.graphpad.com/scientific-software/prism/
CTAn	Bruker	https://www.bruker.com/en/products-and-solutions/microscopes/3d-x-ray-microscopes/xrm-software
CELLQuest Pro software	BD Biosciences	https://www.bdbiosciences.com/zh-cn/products/software
ModFit LT 4.1 software	BD Biosciences	N/A
Scikit-learn	Python 3.6.9	v0.24.1
Seurat	R 4.0	v4.0.3
AUcell	R 4.0	v1.16.0
GSEABase	R 4.0	v1.56.0
BD Rhapsody analysis pipeline	BD Biosciences	v1.9.1

**Other**		

Sequence data, analyses, and resources related to the scRNA sequencing of hADSCs	This paper	N/A
Illumina NextSeq 2000	Illumina	Cat#20038897
BD Rhapsody™ Single-Cell Analysis System	BD Biosciences	Cat#633701
AMPure XP Beads	Beckman Coulter	Cat#A63881
NextSeq 1000/2000 P2 Reagents V3	Illumina	Cat#20046813

### Resource availability

#### Lead contact

Further information and requests for resources and reagents should be directly to and will be fulfilled by the Lead Contact, Y. James Kang (ykang7@uthsc.edu).

#### Material availability

This study did not generate new unique reagents.

### Experimental model and subject details

Detailed methods of this study are provided and include the following:

#### Human adipose-derived stem cells culture, cryopreservation and resuscitation

Adipose tissue suspension was obtained from liposuction plastic surgery and digested with collagenase (Gibco, 17100-017) at 37°C for 1 h (with gentle shaking) then centrifuged to collect primary human adipose-derived mesenchymal stromal cells (hADSC). hADSCs were cultured in αMEM (Gibco, 41061) + 10% FBS (NCT, NCT-HK023) or in chemically-defined IL media (Innolife, 09-RM-HH01A) medium (37°C, 5% CO2) for 20–36 h after seeding andthe medium was changed every 3 d thereafter. The cells were digested with 1×Tryple Express (Gibco, 12604-021) when they reached 70–80% confluence and passaged at 5,000 cells/cm2 density. The solvent used for cryopreservation was CryoPur-D (OriGen Biomedical, CD-50). Frozen samples were rapidly thawed in a water bath at 37°C in continuous agitation, cells were resuspended in pre-warm 1×DPBS, centrifuged at 400 g for 5 min and washed twice with 1×DPBS. Finally, hADSCs were counted and used in other experiments.

This study was conducted in accordance with the established ethical guidelines and approved by the research ethics committee of the Tianjin First Central Hospital, Tianjin, China. Human adipose tissues were surgically obtained from 5 healthy donors at Tianjin First Central Hospital (Table S2). Informed consent was obtained from all volunteers. All studies were conducted under a protocol approved by The Stem Cell Research and Development Center, Tasly Pharmaceutical Co. Ltd.

#### Animal and treatment

NCG mice were purchased from GemPharmatech and all animal protocols were approved by the Institutional Animal Care and Use Committee, Experimental Animal Center. All mice were housed in standard SPF facility with a temperature between 18 and 23°C, a humidity of 40–60%, and a 12 h light-dark cycle. Eight-to-ten-week-old male and female NCG mice were used in this study. The number of mice used in each experiment was indicated, respectively. Mice were randomly assigned into groups. For the injection, 1×10^6^ hADSCs were resuspended in saline and infused into each NCG mouse via tail vein slowly (about 10 s) using a 29-gauge needle.

### Method details

#### Sterility test

Samples were tested by the membrane filtration method under test for sterility, according to the Pharmacopoeia of the People’s Republic of China (2020) Edition. This assay was performed in aseptic conditions. Turned on the germs collector, and filtered about 100mL of 0.9% saline to wet the steritailin which is a cellulose nitrate filter having a nominal pore size not greater than 0.45 μm and a diameter of approximately 50 mm. Then each of the samples was filtered onto a steritailin. After filtration of the samples, the membrane was washed twice by filtering 300 mL of 0.9% saline through it. Two different media were used: Fluid Thioglycollate Medium, to detect anaerobic and aerobic bacteria, and Tryptic Soy Broth (TSB), which is a soybean casein digest medium to detect fungi and aerobic bacteria. A negative control was included inoculating 1 mL of 0.9% saline for each medium. Then add 100mL Fluid Thioglycollate Medium to the two steritailins separately, 100mL TSB to the other steritailin. These inoculated media were incubated under the conditions recommended for sterility tests: one Fluid Thioglycollate Medium at 30–35°C and the others at 20–25°C, cultured for 14 days. Three types of culture media were evaluated daily to detect the presence of microbial growth. If microbial growth appears after 14 days, then the medium will show turbidity.

#### Micro-*CT* examination of pulmonary vasculatures

Mice were anesthetized with avertin and euthanized by cutting off the abdominal aorta. The lung was perfused with 0.9% saline through the right ventricle, followed by perfusion using 5 mL of previously prepared Microfil (2.5 mL MV Compound +2.5 mL MV Diluent +4% MV-Curing Agent, Flow Tech Inc, MV-120). The lung was left for 30 min for the contrast agent to polymerize and was harvested and fixed in a 10% formalin solution. The micro vessels in lungs were imaged using a micro-CT SkyScan1276 (BRUKER), with settings as follows: 50 kV, 180 μA, pixel size 3.05 μm, 0.2-degree rotation step, and 180-degree rotation. The CT section images were captured using a CTAn software to reconstruct three-dimensional images.

#### Histological examination

The lung was harvested immediately after perfusion with 0.9% saline, following the procedure described above. The visual pathological observation was made, and the tissues were fixed in 10% formalin solution over 2 days. The samples underwent dehydration in gradient ethanol and then paraffin embedded. A serial of 4 μm sections were made from each block.

The hematoxylin-eosin (H&E) staining was performed according to a general laboratory procedure (Cardiff et al., 2014). The slides were transferred in xylene for 10 min and then in four serials of changes of ethanol (100%, 95% for 10 min per change and 85%, 75% for 5 min per change). Afterwards, the slides were placed in hematoxylin solution for 10 min and 0.5% hydrochloric acid-alcohol for 3 s, followed by 0.5% ammonium hydroxide for 2 min at room temperature. After rinsing with distilled water, the slides were stained with eosin solution for 3 min. All the slides were added with a drop of permount mounting medium and sealed with coverslips. Martius Scarlet Blue (MSB) stain for fibrin was performed by using a commercial kit (GENMED, GMS80125.1) following the manufacturer’s instructions.

#### Coagulation tests

Mice were anesthetized with avertin, and blood (0.5–1.0 mL) was collected via angular vein using a plastic tube containing 0.11 M sodium citrate (1:9, v: v). The samples were gently mixed and centrifuged at 3,000 *g* for 10 min. After collecting the serum, the prothrombin time (PT), the activated partial thromboplastin time (APTT), the thrombin time (TT), and the fibrinogen (FIB-C) concentrations were determined automatically using an automated blood coagulation analysis system (Compact Max, Stago) following the manufacturer’s protocols.

#### Phenotypic analysis by flow cytometry

The hADSCs of passages 3 or 5 were collected with 1×Tryple Express and centrifuged at 400 g for 5 min. After washing twice with 1×DPBS, cells were resuspended and incubated with pre-labelled antibodies for 15 min at room temperature. After two washes with 1×PBS, cells were resuspended in 300 μL 1×PBS and analyzed using a flow cytometer (BD FACSCalibur). Histograms were generated using the CELLQuest Pro software (BD Biosciences). The antibodies used were as follows: FITC Mouse IgG1,k,Iso-type Ctrl (FC) (BioLegend, 400110), FITC anti-human CD34 (BioLegend, 343504), FITC anti-human CD45, (BioLegend, 304006), FITC anti-human CD11b (BioLegend, 301330), FITC anti-human HLA-DR (BioLegend, 307604), FITC anti-human CD73 (BioLegend, 344016), FITC anti-human CD90 (BioLegend, 328108), APC Mouse IgG1,k,Isotype Ctrl (BioLegend, 400120), APC anti-human CD19 (BioLegend, 363006), PE Mouse IgG1,k,Isotype Ctrl (FC) (BioLegend, 400114), PE anti-CD105 (Endoglin) (BioLegend, 800504).

#### Cell cycle analysis

The cells of passages 3 or 5 were digested with 1×Tryple Express (Gibco, 12604-021) and centrifuged at 400 *g* for 5 min. The cell pellet was washed twice with 1 mL pre-cooled 1×DPBS and resuspended to a density of 1×10^6^ cells/mL. After adding pre-cooled 95% ethanol solution, cells were mixed thoroughly and fixed at 4°C for 2 h. Then, centrifugation was done at 400 *g* for 5 min to precipitate the cells. Propidium iodide solution (4A Biotech, FXP0211) was added to cell samples, and cell precipitation was resuspended and incubated at 37°C for 30 min in dark. After washing twice with 1×PBS, the cells were resuspended in 1×PBS, and the cell cycle was detected using a flow cytometer (BD FACSCalibur) and analyzed by ModFit LT 4.1 software.

#### Cell viability analysis

The cells of passages 3 or 5 were harvested and suspended in 200 μL Binding Buffer (Invitrogen, BMS500FI-300) to a final density of 3×10^5^ cells/mL. Cell suspension (195 μL) was incubated with 5 μL Annexin V-FITC (Invitrogen, BMS500FI-300) for 10 min at room temperature. After a centrifugation, the cells were washed with 200 μL Binding Buffer and resuspended in 190 μL Binding buffer, then incubated with 10 μL Propidium Iodide (20 μg/mL, Invitrogen, BMS500FI-300). Cells were then analyzed using a flow cytometer (BD FACSCalibur).

#### Determination of cell growth kinetics

Cells of passages 3 or 5 were seeded on 96-well microplates with a density of 1000, 2000, 4000, 8000, 16000, or 32000 cells/well. After incubating at 37°C for 4 h, the media were discarded and 110 μL CCK-8 solution (DMEM/F12 without phenol (Gibco, 11039-021): CCK-8 (MCE, HY-K0301) = 10:1) was added into each well. After 2 h, OD at 450 nm was measured using a multifunction microplate reader (Tecan). In a parallel experiment, cells of passages 3 or 5 were plated in 96-well microplates at a density of 1×10^4^ cells/well. The cells were counted each day until the 8th day. Growth curves were plotted using the mean values, and the population doubling time was calculated from the growth curve.

#### Determination of the differentiation potency of hADSCs

##### Osteogenic differentiation

When cultures reached 80–90% confluence, cells were incubated in osteogenic media (Cyagen, HUXMD-90021). The media were replaced every 2 days for 3 weeks, at which time a significant calcium deposit was observed under inverted microscope. The cells were then washed and fixed in 4% paraformaldehyde solution (Beyotime, P0099) and stained by Alizarin Red S (Cyagen, HUXMD-90021) at room temperature for 5 min. After being washed with 1×PBS for three times, images were taken using an inverted phase-contrast microscope (Olympus, CKX53).

##### *Chondrogenic* differentiation

Cells of passages 5 were digested and inoculated into 0.1% gelatin-coated 6-well microplates. When cultures reached 80–90% confluence, cells were induced in chondrogenic medium (Cyagen, HUXMD-90041). The media were replaced every 2–3 days until 2 weeks. The cells were then washed by 1×PBS and fixed in 4% paraformaldehyde solution (Beyotime, P0099) and stained with Alcian blue (Cyagen, HUXMD-90021) at room temperature for 30 min. After washing with 1×PBS for three times, images were taken using an inverted phase-contrast microscope (Olympus, CKX53).

##### Adipogenic differentiation

When cultures reached 100% confluence, cells were incubated in adipogenic medium A (Cyagen, HUXMD-90031) for 3 days, then switched to 2 mL adipogenic medium B (Cyagen, HUXMD-90031) for 1 day. After repeating 3–5 times, the cells were cultured continually in medium B for 4–7 days until lipid droplets appeared. The cells were then washed and fixed using 4% paraformaldehyde solution (Beyotime, P0099) and stained with 0.5% Oil Red O (Cyagen, HUXMD-90031) at room temperature for 20 min. After 1×PBS washing for three times, images were taken using an inverted phase-contrast microscope (Olympus, CKX53).

#### Single cell RNA-seq library generation and sequencing

Single cell RNA-seq library construction of human ADSCs was performed with the BD Rhapsody™ Single-Cell Analysis System (BD Biosciences, 633701), following the manufacture’s guidelines. First, we used the BD Rhapsody™ Scanner to detect the density and viability of hADSCs, and then use the sample calculator function of the scanner to obtain stock cell and buffer volumes to prepare a cell suspension of 650 μL. Next, the hADSCs were loaded on a simple cartridge workflow that contained two hundred thousand microwells. Upon cell lysis, the mRNA content of each cell was captured by probes via polyA/polyT on BD Rhapsody beads that have the same cell label (CL) and a variety of unique molecular identifier (UMI). Subsequently, the first-strand cDNA was reverse-synthesized using oligo (dT) beads. The first-strand cDNAs that were extended through the random primers were eluted into the new tubes without beads, thereafter the products of random primer extension (RPE) were amplified by 13 cycles. Then, 2 nM of the RPE of amplified products was used as the template for PCR with Illumina-sequencing primers and a unique index primer (BD Biosciences, 633801) for an additional 8 cycles. Finally, the new amplified products were purified to obtain the single cell sequencing library that had a unique index label. The library fragments had an average length of approximately 500 bp. Sequencing was performed on Illumina NextSeq 2000 (Illumina, 20038897).

#### Single cell RNA-seq data pre-processing and quality control

Raw sequencing data was demultiplexed and converted to fastq format by using bcl2fastq v2.20 from Illumina. BD Rhapsody analysis pipeline v1.9.1 (BD Biosciences) was used for cell barcode identification, read alignment, and UMI quantification with default parameters. Briefly, filtered R2 reads were aligned to the human reference genome GRCh38 using STAR v2.7.6a, then expression read counts for each gene in all samples were collapsed and adjusted to unique molecular identifier (UMI) counts using recursive substitution error correction (RSEC). Putative cells were identified from background noise using second derivative analysis of all RSEC-adjusted UMI counts. The resulting output is a gene expression matrix with gene identities as columns and cell indices as rows.

#### Quality metrics of samples

RSEC-adjusted UMI count matrices were imported to R 4.1.0. and gene expression data analysis was conducted using the Seurat package 4.0.3 (Butler et al., 2018; Hafemeister and Satija, 2019; Stuart et al., 2019). After identification of singlets, outlier cells were excluded from downstream analyses using the median absolute deviation (MAD) method. Cells with more than 3MAD from the median of mitochondria reads percentage, less than 3MAD from the median of expressed genes, or less than 3MAD from the median of UMI count were considered as outliers. To eliminate confounding effects, such as cell cycle phases, sequencing depth and mitochondria percentage, Seurat was used to regress out the mentioned effects from analysis.

#### Dimensional reduction

To obtain two-dimensional projections of the population’s dynamics, principal component analysis (PCA) was firstly run on the normalized gene-barcode matrix of the top 2,000 most variable genes to reduce the number of feature dimensions using Seurat. The top 2,000 most variable genes were identified based on their mean and dispersion as described by Macoscko et al. (Macosko et al., 2015). After running PCA, uniform manifold approximation and projection (UMAP) (Mcinnes and Healy, 2018) was performed based on top 30 principle components (PCs) to further reduce these components to visualize cells in a two-dimensional space. The number of PCs was selected based on an elbow plot.

#### Cell clustering and subpopulation identification

To uncover similarities and variations between cell clusters across conditions/groups, canonical correlation analysis (CCA) was performed to align cells from different samples into a subspace with the maximal correlation. When clustering cells, it is recommended to regress out confounding factors, including the percentage of mitochondrial RNA, cell cycle and experimental batch effects. After regressing out confounding factors, an unsupervised graph-based clustering was performed to group cells for the clustering analysis (Levine et al., 2015) and cluster-specific marker genes were also identified using Seurat software.

#### Identification of differentially expressed genes (DEGs)

Differential gene-expression analysis was performed using the Wilcox rank sum test from Seurat. Genes were identified as significantly differentially expressed genes with false discovery rate (FDR) < 0.05 and at least a log-fold change of 0.25 in expression between clusters.

#### Gene function analysis

Gene enrichment analysis was conducted on gene sets from the Kyoto Encyclopedia of Genes and Genomes (KEGG) (Kanehisa and Goto, 2000) database and Gene Ontology (GO) (Ashburner et al., 2000) using R package ClusterProfiler v.4.1.1. Additionally, the embolic risk score was assigned to each cell by using “AddModuleScore” function according to the related gene sets from Reactome database (i.e., common pathway of fibrin clot formation, extrinsic pathway of fibrin clot formation, formation of fibrin clot and intrinsic pathway of fibrin clot formation).

#### Functional clustering based on canonical pathway scores

To convert sparse gene expression matrix to pathway score matrix, canonical pathway scores were estimated using ssGSEA (Barbie et al., 2009), AUCell (Aibar et al., 2017) and “AddModuleScore” function of Seurat with default settings. Then dimensional reduction and visualization were conducted based on the pathway score matrix as described above.

#### Derivation of a predictive model for embolism risk

After cell cluster analysis, we used gene expression in stem cells as feature vectors to build a predictive model. We randomly selected 70% pro-embolic cells (3266) and non-embolic cells (2327) from A2105C2P5 and A2105C3P5, respectively, as the training set. The remaining cells of A2105C2P5 and A2105C3P5 were regarded as our test 1 set. We also took pro-embolic and non-embolic stem cells from A2105-P3, A2013-P5 and A2106-P3 as test 2, test 3 and test 4 set, respectively (Table S1). Then, we randomly chose 70% cells of the training set to be the actual training set and the remaining 30% cells to be the validation set to select feature genes and train models. Ten SVM-RFEs models with different regularization parameter (C) value (C = 0.2, 0.6, 0.8, 1.0, 1.2, 1.6, 2.0, 2.2, 2.6 and 3.0) and 10-fold cross validation were used in feature selection. We used selected features and set different C values (C = 0.0001, 0.0005, 0.001, 0.002, 0.004, 0.008, 0.02, 0.05, 0.2, 0.6, 1.2, 1.8, 2.4 and 3) to train fourteen linear SVM models. After evaluating test accuracy, precision, recall and F1 score of these models in four test sets, linear SVM with C = 0.2 had the best performance, and was determined as the final predictive model. All machine learning processes were implemented using scikit-learn 0.24.2 in python 3.6.9.

#### A mathematical model to predict embolic risk

A mathematical model for calculating the embolic risk of single hADSCs was developed through the analysis of 13 key genes and their weighted coefficient. First, we input the training set T = {(x_1_, y_1_), (x_2_, y_2_), …, (x_n_, y_n_); where x_i_ is the eigenvector representing cell i, yi ∈{+1, −1) representing embolic and non-embolic label, i = 1, 2, … n. Then, we use the linear SVM algorithm to classify embolic and non-embolic hADSCs. The SVM needs to solve the normal vector **w** and the displacement value b to obtain uniquely optimal separating hyperplane, based on the assumption that the training data are linearly separable and **w** and b must satisfy the inequality (Equation 4).

(Equation 4) $$y_{j}\left(w\cdot x_{j}+b\right)\geq 1$$

Where $\left(x_{j},y_{j}\right)$ is the cell as a support vector. However, there is almost no completely linearly separable training data in practice. So, we solve the **w** and b by the following optimization problem (Equation 5).

(Equation 5) $$\min_{w,\;b,\xi_{i}}\frac{1}{2}{\left|\left|w\right|\right|}^{2}+C\overset{m}{\underset{i=1}{\sum }}\xi_{i}\;$$

$$\text{s}.\text{t}.\;y_{j}\left(w\cdot x_{j}+b\right)\geq 1-\xi_{i}$$

$$\xi_{i}\geq 0,\;i=1,\;2,\;3,\;\ldots \;,\;n$$

Where $\xi_{i}$ is a “relaxation variable”, which is determined by the Hinge loss function. Each cell has a relaxation variable which represents the degree to which the cell does not meet the linearly separable assumption. The C is the penalty parameter that controls the complexity of the model. Second, we set C > 0 and constructed and solved convex quadratic programming problem using Equation (6)

(Equation 6) $$\min_{\alpha }\frac{1}{2}\overset{n}{\underset{i=1}{\sum }}\overset{n}{\underset{j=1}{\sum }}\alpha_{i}\alpha_{j}y_{i}y_{j}\left(x_{i}\cdot x_{j}\right)-\overset{n}{\underset{i=1}{\sum }}\alpha_{i}$$

$$\text{s}.\text{ t}.\overset{N}{\underset{i}{\sum }}\alpha_{i}y_{i}=0$$

$$\text{0}\leq \;\alpha_{i}\;\leq \text{C, i}=1\text{, }2...\text{ n}$$

Where  α  is the Lagrange multiplier. The optimal α^∗^ = (α_1_^∗^, α_1_^∗^ … α_n_^∗^) ^T^ was calculated using Sequential minimal optimization algorithm. Secondly, we calculated the normal vector **w** of the optimal separating hyperplane which can significantly distinguish the embolic and non-embolic cells using Equation (7)

(Equation 7) $$w=\sum_{i=1}^{n}\alpha_{i}^{*}y_{i}\left(x_{i}\cdot x_{j}\right)$$

Select a component of $\alpha^{*}$: $\alpha_{j}^{*}$ to satisfy the condition 0 < $\alpha_{j}^{*}$< C, and calculate b using Equation (8)

(Equation 8) $$\text{b}=y_{i}-\sum_{i=1}^{n}\alpha_{i}^{*}y_{i}\left(x_{i}\cdot x_{j}\right)$$

After obtaining **w** and b, the optimal separated hyperplane can be expressed as Equation (9)

(Equation 9) w⋅x+b=0

Because the normal vector of separated hyperplane **w** is the weighted coefficient vector of 13 key genes. So, we quantified the embolic risk of each hADSC using Equation (10)

(Equation 10) $$\text{RS}=1+e^{-\sum_{i=1}^{N}w_{i}*G_{i}}$$

Where $w_{i}$ is the coefficient parameter of *i*th gene and is also the *i*th element of the weighted coefficient vector **w**, $G_{i}$ is the expression of *i*th gene in this cell, N is the number of key genes.

### Quantification and statistical analysis

Graphs and statistical tests were generated using GraphPad Prism v.9. Sample sizes were chosen based on previous studies and no statistical methods were used to predetermine sample size. Mice for each experimental group were assigned randomly and the investigators were blinded to the experiments before analysis. Data was analyzed using Student’s unpaired two-tailed t-test when comparing two conditions and ANOVA when comparing multiple conditions. Sample size and treatment condition for each experiment are indicated in the figure legends. Each data point represents a biological replicate; data are mean ± s.e.m. Statistical significance of difference was considered when ∗p < 0.05.

## Data Availability

Single-cell RNA-seq data have been deposited and accession numbers are listed in the key resources table. This paper does not report original code. DOIs are listed in the key resources table. Any additional information required to reanalyze the data reported in this paper is available from the lead contact upon request.
