# Supplementary material for: Transcriptomic heterogeneity of cultured ADSCs corresponds to embolic risk in the host
Source: iScience. 2022 Aug 4;25(8):104822. doi: 10.1016/j.isci.2022.104822 (PMC9389247; doi:10.1016/j.isci.2022.104822)
Supplement: Document S1. Figures S1–S6 and Tables S1–S4 [file mmc1.pdf]

iScience, Volume *25*

Supplemental information

Transcriptomic heterogeneity of cultured ADSCs corresponds to embolic risk in the host

Kaijing Yan, Jinlai Zhang, Wen Yin, Jeffrey N. Harding, Fei Ma, Di Wu, Haibo Deng, Pengfei Han, Rui Li, Hongxu Peng, Xin Song, and Y. James Kang

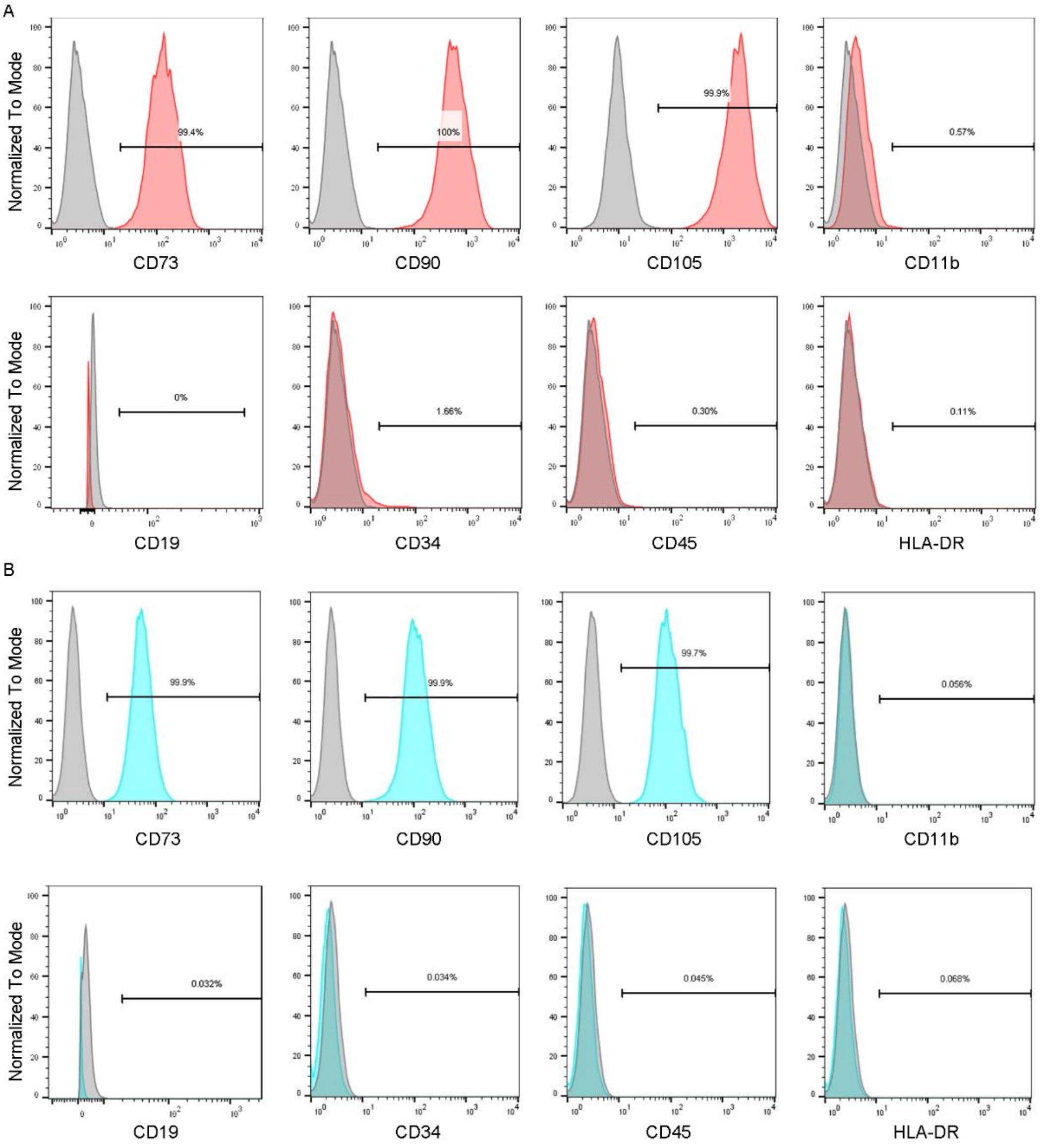

Figure S1. Phenotypic analysis of hADSCs by flow cytometry, related to STAR Methods.

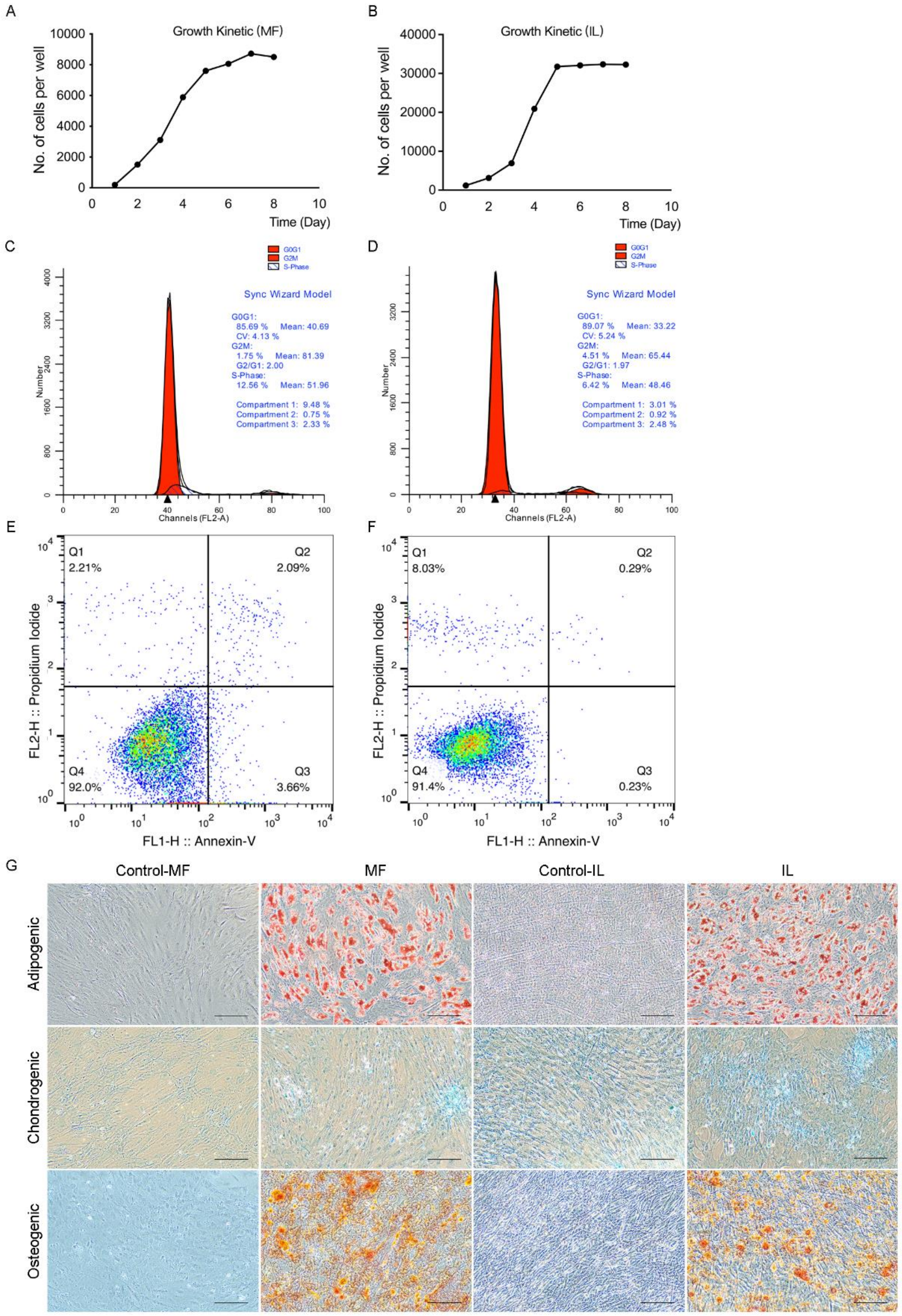

Figure S2. Growth curves, cell cycle, cell viability and differentiation of hADSCs, related to STAR Methods.

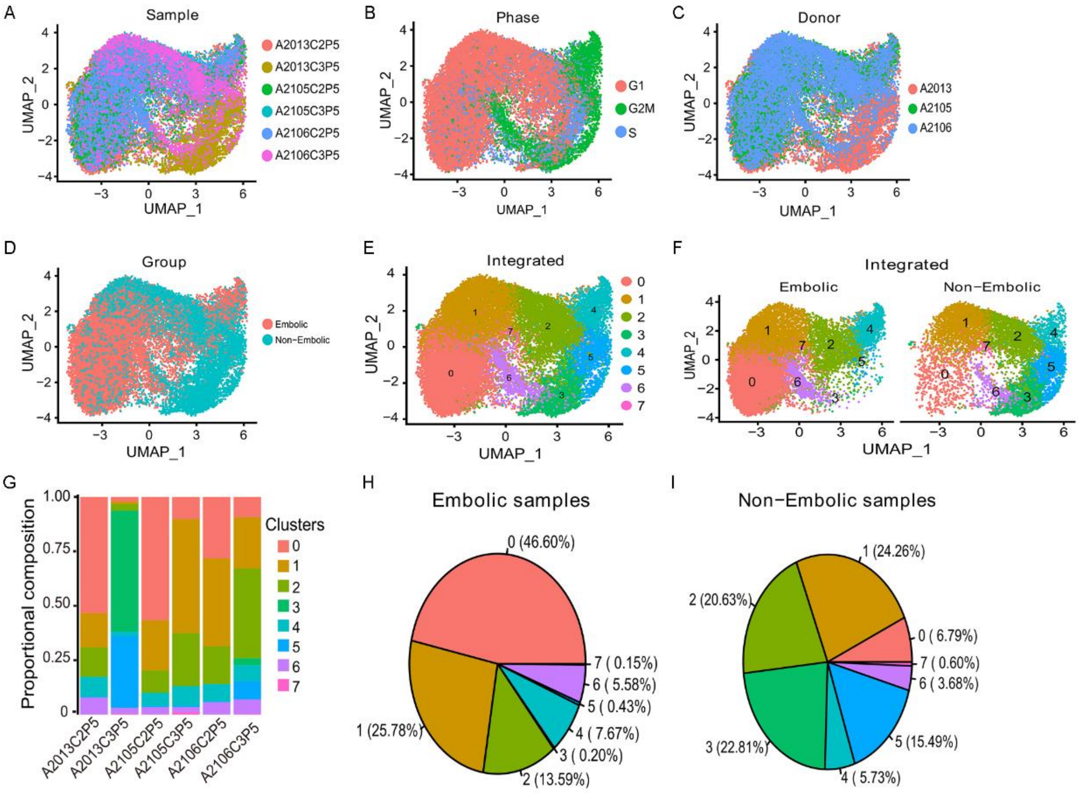

Figure S3. Heterogeneity of hADSCs revealed by UMAP analyses, related to STAR Methods.

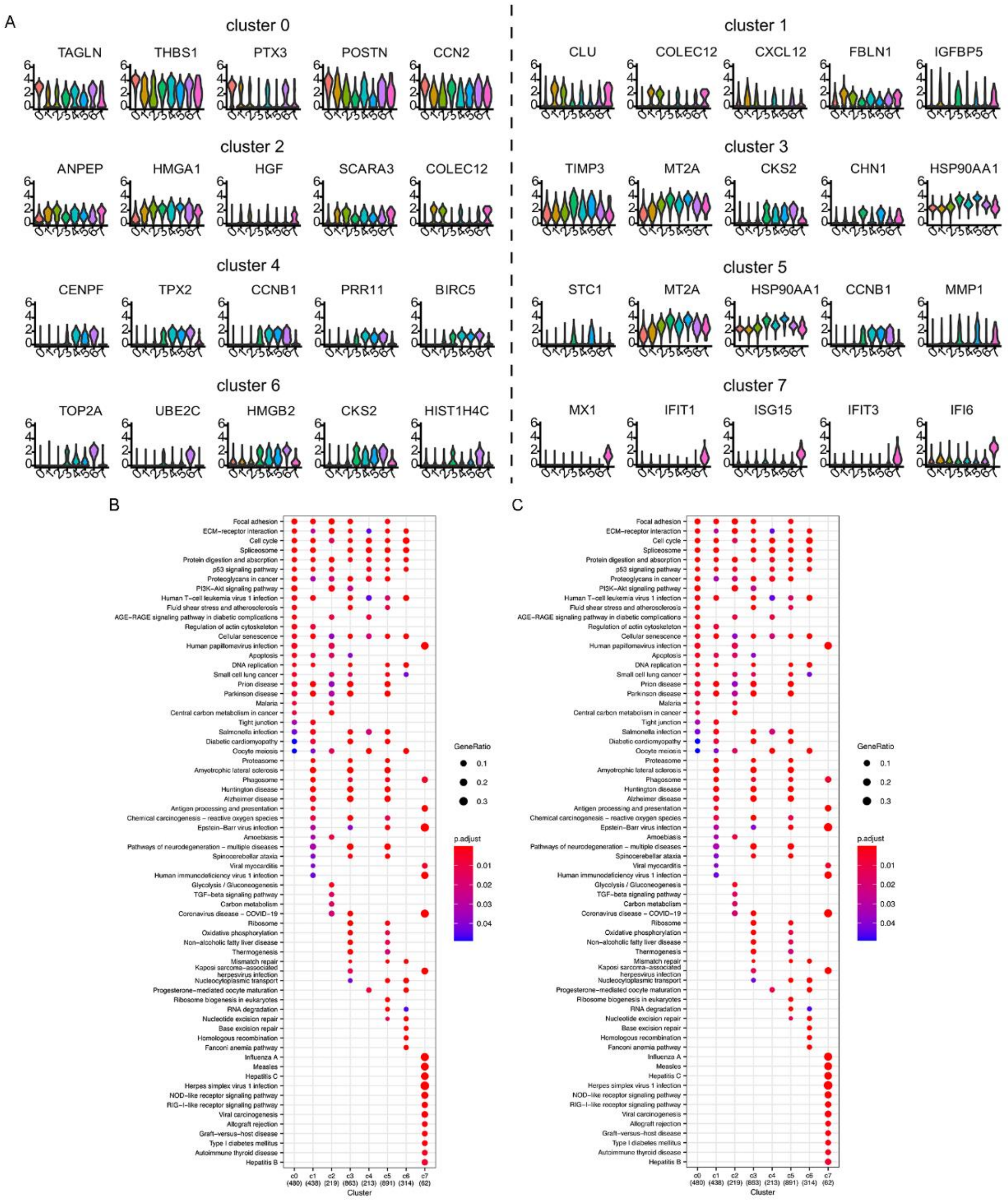

Figure S4. Transcriptomic analysis of UMAP clusters for hADSCs pooled from two different culture conditions, related to STAR Methods.

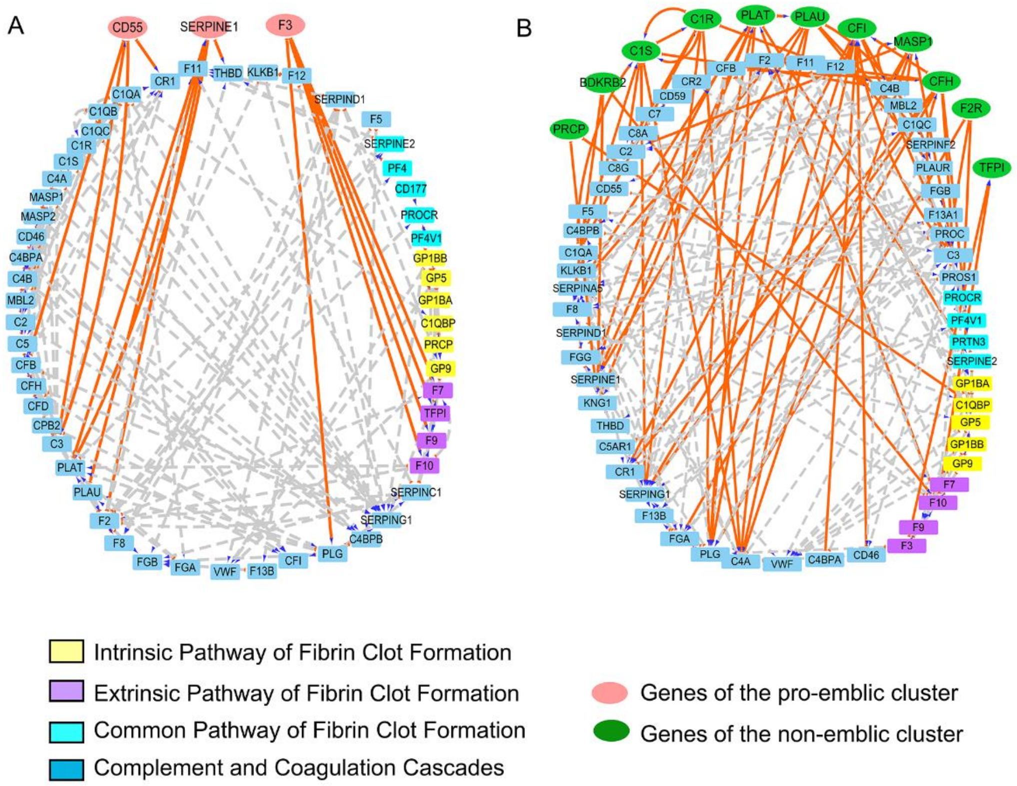

Figure S5. PPI networks of embolism related genes and pathways, related to Figure 3.

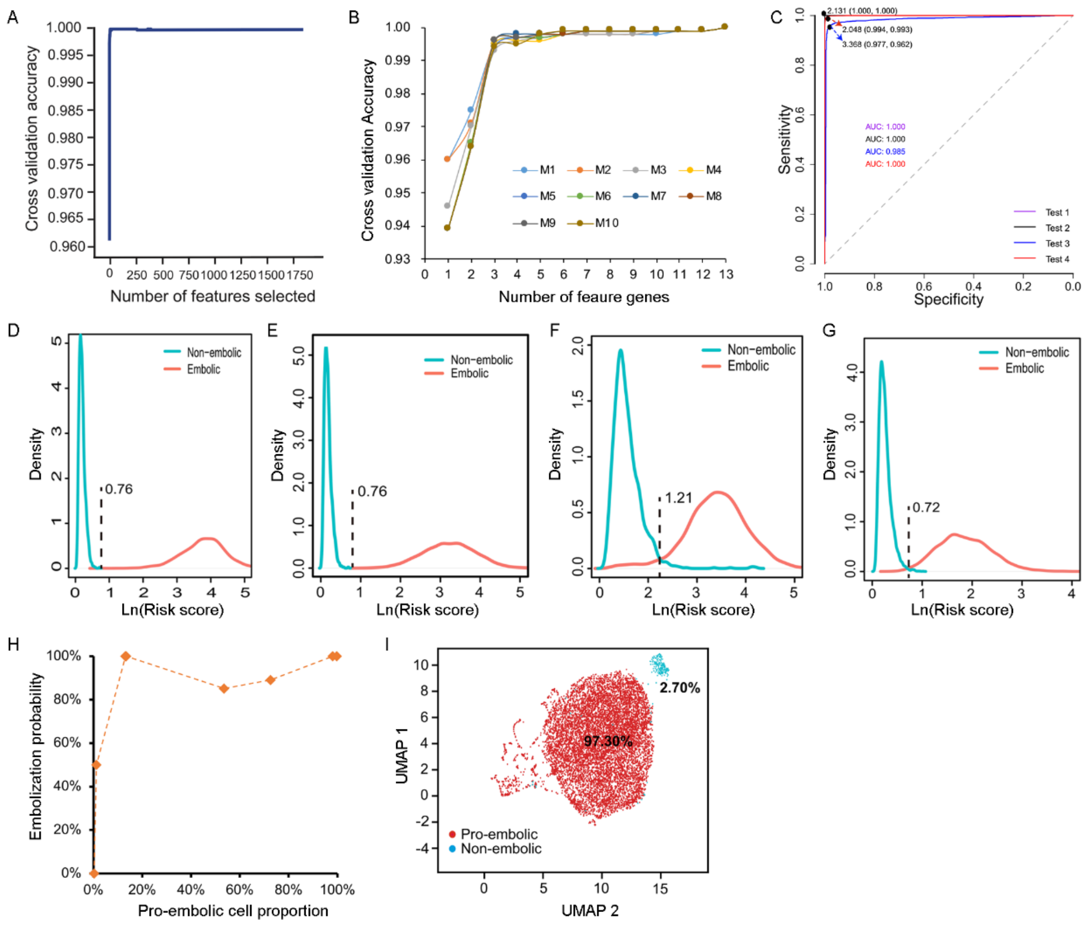

Figure S6. Development of a mathematical model for predicting embolism risk, related to Figure 3.

Table S1. Accuracy of models in train and test datasets, related to STAR Methods.

Model (C value)	Train dataset	Test 1	Test 2	Test 3	Test 4
		Similar to train set	Different generation	Different donor	Different donor and generation
3	1.00	1.00	1.00	0.97	0.95
2.4	1.00	1.00	1.00	0.97	0.95
1.8	1.00	1.00	1.00	0.97	0.95
1.2	1.00	1.00	1.00	0.97	0.95
0.6	1.00	1.00	1.00	0.97	0.95
0.2	1.00	1.00	1.00	0.97	0.95
0.05	1.00	1.00	1.00	0.97	0.89
0.02	1.00	1.00	1.00	0.97	0.92
0.008	1.00	1.00	1.00	0.95	0.8
0.004	1.00	1.00	1.00	0.96	0.78
0.002	1.00	1.00	1.00	0.96	0.77
0.001	1.00	1.00	1.00	0.96	0.71
0.0005	1.00	1.00	1.00	0.97	0.71
0.0001	1.00	1.00	1.00	0.95	0.67

Table S2. Donor information, related to STAR Methods.

Number in dataset	histologic origin	Sex	Race/Ethnicity	Volume
**A20-13**	Abdominal subcutaneous adipose tissue	F	Chinese	100 mL
**A21-05**	Abdominal subcutaneous adipose tissue	M	Chinese	100 mL
**A21-06**	Adipose tissue of the inner thigh	F	Chinese	100 mL
**SA5**	Adipose tissue of the inner thigh	F	Chinese	80 mL
**SA19**	Adipose tissue of the inner thigh	F	Chinese	80 mL

Table S3. Sample and data used for model development, related to STAR Methods.

Sample ID	Donor	Culture	Generation	Used for	Phenotype of cells
A2105C2P5(70%)	A2105	MF	P5	Train	Pro-embolic
A2105C3P5(70%)	A2105	IL	P5	Train	non-embolic
A2105C2P5(30%)	A2105	MF	P5	Test 1	Pro-embolic
A2105C3P5(30%)	A2105	IL	P5	Test 1	non-embolic
A2105C2P3	A2105	MF	P3	Test 2	Pro-embolic
A2105C3P3	A2105	IL	P3	Test 2	non-embolic
A2013C2P5	A2013	MF	P5	Test 3	Pro-embolic
A2013C3P5	A2013	IL	P5	Test 3	non-embolic
A2106C2P3	A2106	MF	P3	Test 4	Pro-embolic
A2106C3P3	A2106	IL	P3	Test 4	non-embolic

Table S4. Genes of pro-embolic hADSC subpopulation identified by functional clustering procedure, related to Figure 3.

Gene symbol	Function in embolism	Coagulation-related pathway	MF medium		IL medium	
			C0_exp	C3_exp	C0_exp	C3_exp
SERPINE1	pro-embolic	Complement and coagulation cascades	up-regulated	up-regulated	non-significant	up-regulated
F3	pro-embolic	Extrinsic Pathway of Fibrin Clot Formation<br>Complement and coagulation cascades	up-regulated	non-significant	non-significant	up-regulated
CD55	pro-embolic	Complement and coagulation cascades	non-significant	up-regulated	non-significant	non-significant
BDKRB2	anti-embolic	Complement and coagulation cascades	non-significant	non-significant	non-significant	down-regulated
C1R	anti-embolic	Complement and coagulation cascades	down-regulated	down-regulated	non-significant	down-regulated
C1S	anti-embolic	Complement and coagulation cascades	non-significant	non-significant	non-significant	down-regulated
CFH	anti-embolic	Complement and coagulation cascades	down-regulated	down-regulated	non-significant	down-regulated
CFI	anti-embolic	Complement and coagulation cascades	non-significant	non-significant	non-significant	down-regulated
F2R	anti-embolic	Common Pathway of Fibrin Clot Formation<br>Complement and coagulation cascades	down-regulated	down-regulated	non-significant	non-significant
MASP1	anti-embolic	Complement and coagulation cascades	down-regulated	down-regulated	non-significant	down-regulated
PLAT	anti-embolic	Complement and coagulation cascades	down-regulated	down-regulated	non-significant	non-significant
PLAU	anti-embolic	Complement and coagulation cascades	down-regulated	down-regulated	non-significant	non-significant
PRCP	anti-embolic	Intrinsic Pathway of Fibrin Clot Formation	non-significant	non-significant	non-significant	down-regulated
TFPI	anti-embolic	Extrinsic Pathway of Fibrin Clot Formation<br>Complement and coagulation cascades	down-regulated	down-regulated	non-significant	non-significant
